# Potentiometric Study of Carbon Nanotube/Surfactant Interactions by Ion-Selective Electrodes. Driving Forces in the Adsorption and Dispersion Processes

**DOI:** 10.3390/ijms22020826

**Published:** 2021-01-15

**Authors:** Francisco José Ostos, José Antonio Lebrón, María Luisa Moyá, Eva Bernal, Ana Flores, Cristian Lépori, Ángeles Maestre, Francisco Sánchez, Pilar López-Cornejo, Manuel López-López

**Affiliations:** 1 Department of Physical Chemistry, University of Seville, c/Prof. García González 1, 41012 Seville, Spain; fostos@us.es (F.J.O.); lebronjunior@hotmail.com (J.A.L.); moya@us.es (M.L.M.); evabernal@us.es (E.B.); gcjrv@us.es (F.S.); 2 Department of Chemical Engineering, Physical Chemistry and Materials Science, Campus ‘El Carmen’, Faculty of Experimental Sciences, University of Huelva, 21071 Huelva, Spain; a.flopez@hotmail.com (A.F.); a.mvazquez1994@gmail.com (Á.M.); 3 Institute of Physics Enrique Gaviola (IFEG), National Council of Scientific and Technical Research (CONICET), National University of Córdoba (UNC), Córdoba X5016LAE, Argentina; cristianlepori@hotmail.com

**Keywords:** carbon nanotubes, ionic surfactants, potentiometric technique, ion-selective electrodes, adsorption and dispersion

## Abstract

The interaction (adsorption process) of commercial ionic surfactants with non-functionalized and functionalized carbon nanotubes (CNTs) has been studied by potentiometric measurements based on the use of ion-selective electrodes. The goal of this work was to investigate the role of the CNTs’ charge and structure in the CNT/surfactant interactions. Non-functionalized single- (SWCNT) and multi-walled carbon nanotubes (MWCNT), and amine functionalized SWCNT were used. The influence of the surfactant architecture on the CNT/surfactant interactions was also studied. Surfactants with different charge and hydrophobic tail length (sodium dodecyl sulfate (SDS), octyltrimethyl ammonium bromide (OTAB), dodecyltrimethyl ammonium bromide (DoTAB) and hexadecyltrimethyl ammonium bromide (CTAB)) were studied. According to the results, the adsorption process shows a cooperative character, with the hydrophobic interaction contribution playing a key role. This is made evident by the correlation between the free surfactant concentration (at a fixed [CNT]) and the critical micellar concentration, *cmc*, found for all the CNTs and surfactants investigated. The electrostatic interactions mainly determine the CNT dispersion, although hydrophobic interactions also contribute to this process.

## 1. Introduction

Carbon nanotubes (CNTs) are a carbon allotrope synthesized for the first time by Oberlin et al. by pyrolising benzene and hydrogen at 1100 °C [1] and Iijima by using arc-discharge evaporation of carbon [2]. CNTs show a cylindrical nanostructure with single or multiple nanowalls. This structure is defined by a chiral vector (n, m) that specifies the way that the graphene sheet is rolled into a tube [3]. Single-walled (SWCNT) and multi-walled (MWCNT) carbon nanotubes are interesting nanomaterials with exceptional mechanical, thermal, electronic, optical and chemical properties such as high tensile strength, ultra-light weight, special electronic structures, unique absorption, fluorescence and Raman spectra and high chemical and thermal stability. As a consequence of these properties, they show a wide range of applications. For example, they have been used in electronics, polymer composites, energy storage materials, catalysis, gas storage materials, sensors, environment and biomedicine [4,5,6,7,8,9,10,11,12]. These applications are limited due to a low and poor solubility in solution because of the existence of strong Van der Waals forces and π-π stacking interactions among the tubes, which provoke their agglomeration into ropes and/or bundles [13,14,15]. In fact, the energy of the contact between two carbon nanotubes by Van der Waals interactions is about 500 eV/µm [16]. The dispersion of CNTs can be improved by physical (mechanical) and chemical methods [17,18]. Ultrasonication is the most widely used physical method to disperse CNTs in aqueous solution, but unfortunately this technique also produces the fragmentation of the tubes [17]. Chemical methods include covalent and non-covalent approaches [18]. The former consists in the functionalization of the CNTs walls with different chemical groups, increasing their charge and, consequently, decreasing their agglomeration. However, this method can affect the electrical and mechanical properties of nanotubes [19,20]. The latter involves the adsorption of chemical compounds on the CNT walls by either electrostatic or π-π stacking interactions. The honeycomb lattice is preserved and the π bonds of the nanotubes remain undisturbed [21].

Polymers and surfactants are commonly used in the noncovalent approach to exfoliate the CNT bundles and disperse them [18,21,22,23,24,25,26,27]. The ability of polymers to stabilize the dispersion is based on the formation of complexes in which the polymeric chains are wrapped around the tubes. In the case of surfactants, the dispersion of the carbon nanotubes is based on the adsorption of surfactant molecules on the tube surface. Studies about the interactions between surfactants and CNTs suggest three possible mechanisms: (i) the formation of cylindrical micelles at the nanotubes surfaces [28], (ii) the adsorption of hemimicelles onto the tubes [29], and (iii) the random adsorption of surfactant molecules, without a defined structure [30,31]. The dispersing capacity of the surfactants is closely linked to their molecular structure. For example, the gemini surfactants are better CNT dispersants than single-tailed surfactants [26,32] and the presence of aromatic groups in the surfactant molecule structure improves their dispersing ability [33,34]. In addition, it has been shown that the dispersing capacity of a mixed system of cationic and anionic surfactants is greater than that of individual surfactants, obtaining stable dispersions even at high CNTs concentrations as a result of the synergetic effect of the mixture [35].

In a previous paper, the authors demonstrated that a structureless random adsorption of different commercial ionic surfactants on carboxylated SWCNT (SWCNT-COOH) walls takes place, the hydrophobic interactions being the driving force on this process. The electrostatic interactions do not influence on the adsorption process, but they are the driving force of the dispersion phenomenon [36]. In order to investigate the role of the CNT nature (charge and type) and deepen the research of CNT/surfactant interactions, quantitative information about the adsorption of ionic commercial surfactants in non-functionalized SWCNT and MWCNT surfaces as well as at amine functionalized SWCNT (SWCNT-NH_2_) surfaces was obtained. This study was carried out using potentiometric measurements with ion-selective electrodes for the different surfactants used.

The results obtained in this work will contribute to the understanding of the adsorption process of ionic surfactants at CNTs surfaces (CNT/surfactant interactions). To the best knowledge of the authors, this is the first time that a quantification of the interactions of SWCNT, MWCNT, and SWCNT-NH_2_ with single-chained ionic surfactants is provided. They will also shed new light onto the difference between the adsorption of ionic surfactants at the CNT surfaces and the dispersion of these nanostructures by these ionic surfactants. Understanding the driving forces of both processes is important in relation to the wide range of applications of CNTs in which a good dispersion of the carbon nanotubes is essential [4,5,6,7,8,9,10,11,12].

## 2. Results and Discussion

### 2.1. Carbon Nanotube/Surfactant Interactions: Adsorption Process

In order to obtain quantitative information about how the adsorption process of ionic surfactants on CNTs surfaces happens, the interactions of these surfactants with SWCNT, MWCNT and SWCNT-NH_2_ have been studied using potentiometric techniques with ion-selective membrane electrodes. In a previous paper, the authors highlighted the usefulness of these techniques to quantify the interactions of ionic surfactant with SWCNT-COOH [36]. The measurements of electromotive forces (emf) of solutions containing the ionic surfactant in the presence of carbon nanotubes give information of the amount of surfactant which does not interact with the CNTs surfaces. The dependence of the electromotive force on the free surfactant activity is given by the Nernst equation:

(1) $$E=E^{0\prime }-\frac{RT}{z_{i}F}\log a_{i}$$

In all cases, the surfactant concentration was below the critical micellar concentration. Given that the solutions are so diluted, the free surfactant activity can be substituted by the surfactant concentration, [S]_f_. The membrane gave a Nernstian response with the logarithm of the concentration for all the surfactants investigated, with slope values close to 59 mV at 298.1 ± 0.1 K (see Figure S1 in Supplementary Information). The linear plots of emf versus log [S]_f_ are the calibration curves to obtain the free surfactant concentration in a determined solution.

**Figure 1:**
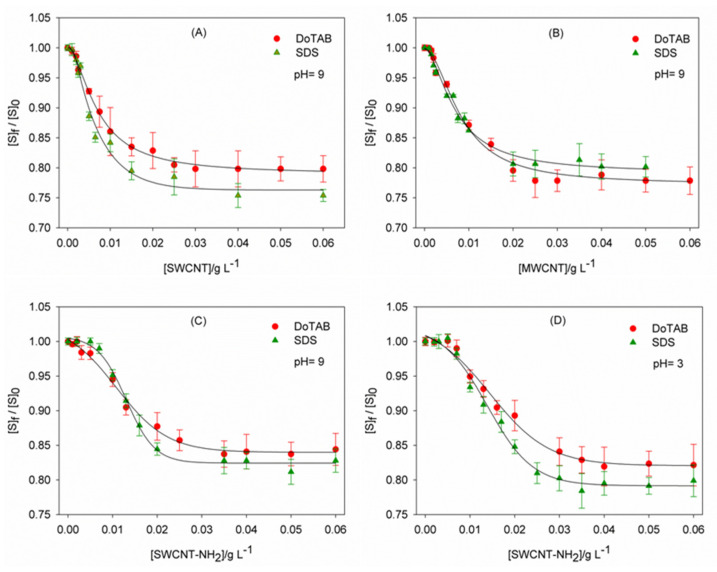
Plots of the relative free dodecyltrimethyl ammonium bromide (DoTAB) and sodium dodecyl sulfate (SDS) concentration versus the carbon nanotubes concentration in buffered aqueous solutions: (**A**) neutral single-walled carbon nanotubes (SWCNT); (**B**) neutral multi-walled carbon nanotubes (MWCNT); (**C**) neutral SWCNT-NH_2_ (pH = 9); and (**D**) positively charged SWCNT-NH_2_ (pH = 3).

**Figure 2:**
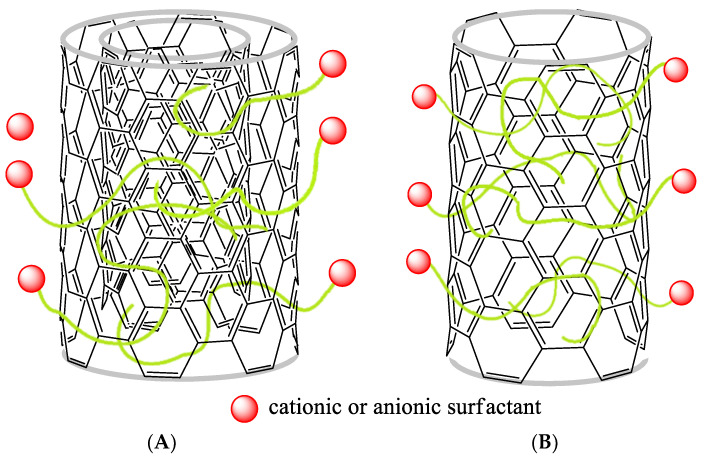
Schematic picture of the adsorption of anionic and cationic surfactants on the MWCNT (**A**) and SWCNT (**B**) surface.

According to the results of the emf measurements, a sigmodal decrease of the relative free surfactant concentration, [S]_f_/[S]_0_ ([S]_0_ = total surfactant concentration), with the CNT concentration is observed for all the CNTs studied (see Figure 1). As is well known, this dependence indicates a cooperative character of the CNT/surfactant interactions, that is, the binding of a surfactant molecule to a carbon nanotube makes the binding of a second surfactant molecule to the same tube more favorable and so on. This result is in accordance with those found in other experimental [36] and simulation studies [37]. As can be seen in Figure 1, the cooperativity of CNT/surfactant interaction is independent of the type of carbon nanotubes (see Figure 1A,B). This observation could indicate that the adsorption of the surfactant molecules occurs mainly on the outer wall of the MWCNT (see Figure 2A). Furthermore, neither the surface charge of the neutral (SWCNT (Figure 1A), MWCNT (Figure 1B), SWCNT-NH_2_ pH = 9 (Figure 1C)) or positive (SWCNT-NH_2_ pH = 3 (Figure 1D)) carbon nanotubes, nor the polar headgroup charge of the ionic surfactants (SDS and DoTAB) influence the adsorption process. These results show that the electrostatic interactions do not play an important role in the adsorption of the ionic surfactants investigated and, as in the case of anionic single-walled carbon nanotubes [36], the mechanism of the adsorption process involves random interactions.

**Figure 3:**
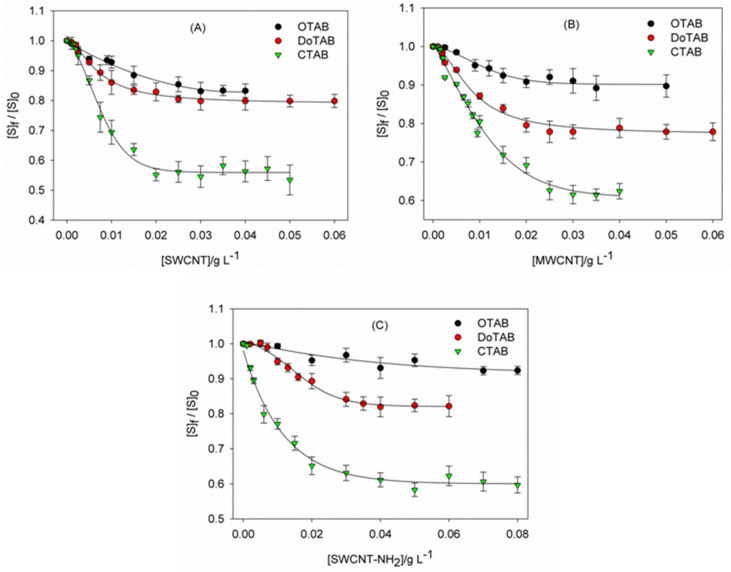
Plots of the relative free surfactant concentration versus the carbon nanotubes concentration in buffered aqueous solutions: (**A**) SWCNT (pH = 9); (**B**) MWCNT (pH = 9); and (**C**) SWCNT-NH_2_ (pH = 3).

This mechanism would imply that the adsorption process takes place through hydrophobic interactions between the hydrocarbon tails of the surfactant molecules and the surface of CNTs, while the hydrophilic head groups are directed outward in contact with the solvent [36,38]. On these bases, one would expect that the adsorption process mainly depended on the length of the hydrocarbon tail of the surfactant monomers. In order to test this hypothesis, and deepen our research, the study of the interactions of three cationic surfactants, with the same head group and different chain length, with various CNTs has been carried out. The results are shown in Figure 3.

**Figure 4:**
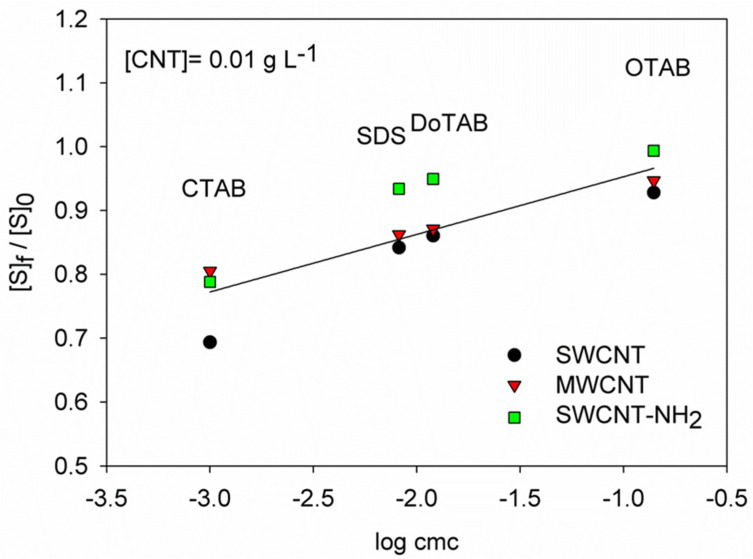
Plot of [S]_f_/[S]_0_, at [CNT] = 0.01 g L^−1^, *versus* log (*cmc*/mol dm^−3^) for all ionic surfactants and CNTs investigated.

As can be seen, a decrease of the ratio [S]_f_/[S]_0_ with the amount of CNT in the aqueous solution is obtained for all the surfactants and carbon nanotubes investigated. The dependence of [S]_f_/[S]_0_ on [CNT] is strongly influenced by the chain length of the ionic surfactant: the longer the hydrocarbon tail, the higher the decrease of [S]_f_/[S]_0_ upon increasing CNTs concentration is, the trend in the relative free concentration being CTAB < DoTAB < OTAB. For a given surfactant, neither the type of the nanotubes nor their charge practically influence the formation of the CNT/surfactant complexes (see Figure 3). These results show that the hydrophobic interactions are the driving force in the adsorption process of the surfactant molecules on the CNTs surfaces, these hydrophobic surfaces attracting the hydrophobic tails of the surfactants. Therefore, the relative free surfactant concentration, for a given [CNT], would be expected to show a correlation with a parameter such as the critical micellar concentration (*cmc*), since the hydrophobic Gibbs energy term is the main contribution to the ΔG_micellization_ and to the *cmc* [39]. As can be seen in Figure 4 and Figure S2 (Supplementary Information), a good linear correlation of [S]_f_/[S]_0_, for [CNT] = 0.01 g L^−1^, with the log *cmc* was observed for all the ionic surfactants [40,41] and CNTs studied.

### 2.2. Carbon Nanotube/Surfactant Interactions: Dispersion Process

**Figure 5:**
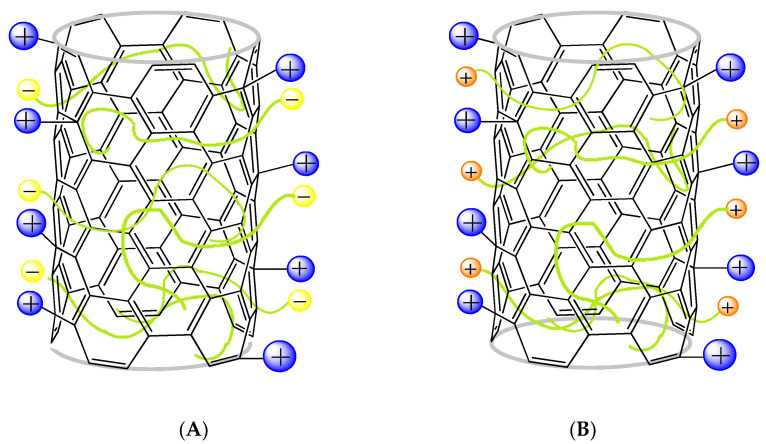
Schematic picture of the adsorption of anionic (**A**) and cationic (**B**) surfactants on the charged SWCNT-NH_2_ surfaces (pH = 3).

As was mentioned above, the disaggregation of the CNTs bundles to obtain a uniform dispersion is essential for the applications of these carbon nanostructures. In regard to the use of surfactants as dispersing agents, the authors demonstrated in a previous study that the dispersion of SWCNT-COOH with ionic surfactants is mainly determined by the electrostatic interactions between the CNT/surfactant complexes [36]. For surfactants with tails of the same length, the one that produces the highest increase in the surface charge of CNTs is the best dispersing agent, since an increment in the electrostatic repulsions among the CNT/surfactant complexes favors the dispersion. In the present work, adsorption of both anionic (SDS) and cationic (DoTAB) surfactants at non-functionalized neutral carbon nanotube (SWCNT and MWCNT) surfaces increases the surface charge of the tubes (see Figure 2) and, consequently, the quality of the dispersion would be the same with any of these additives. However, in the case of the positively charged CNTs (SWCNT-NH_2_ pH = 3), cationic surfactants are the only ones that increase the positive charge of the nanotubes walls (see Figure 5) and, as a result, they would favor their dispersion in solution due to an increase in the electrostatic repulsions.

**Figure 6:**
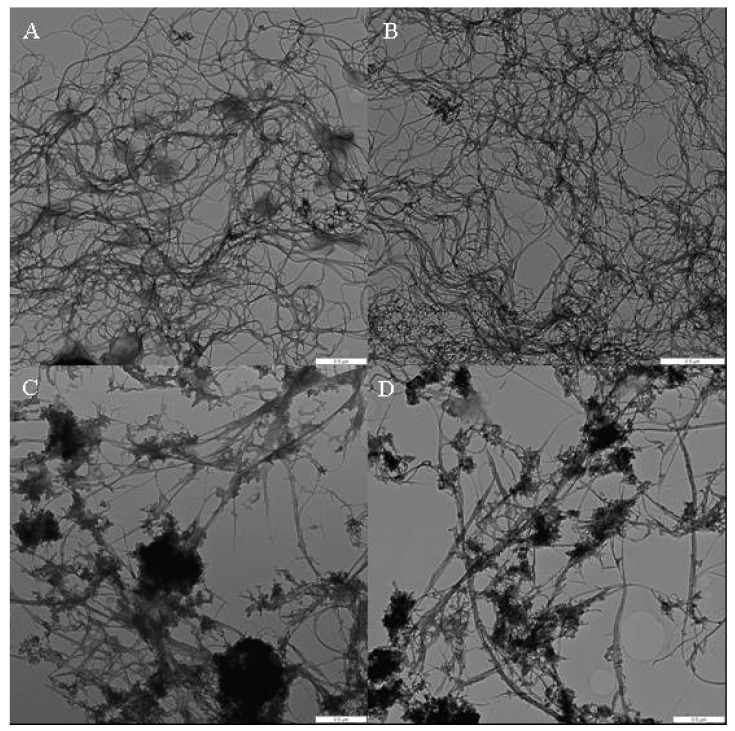
Transmission electron microscopic images of MWCNT/SDS (**A**), MWCNT/DoTAB (**B**), SWCNT/SDS (**C**) and SWCNT/DoTAB (**D**) with scale bars of 0.5 μm. The samples were prepared in a buffer solution Na_2_HPO_4_ 0.01 M/NaCl 0.02 M, pH 9.

**Figure 7:**
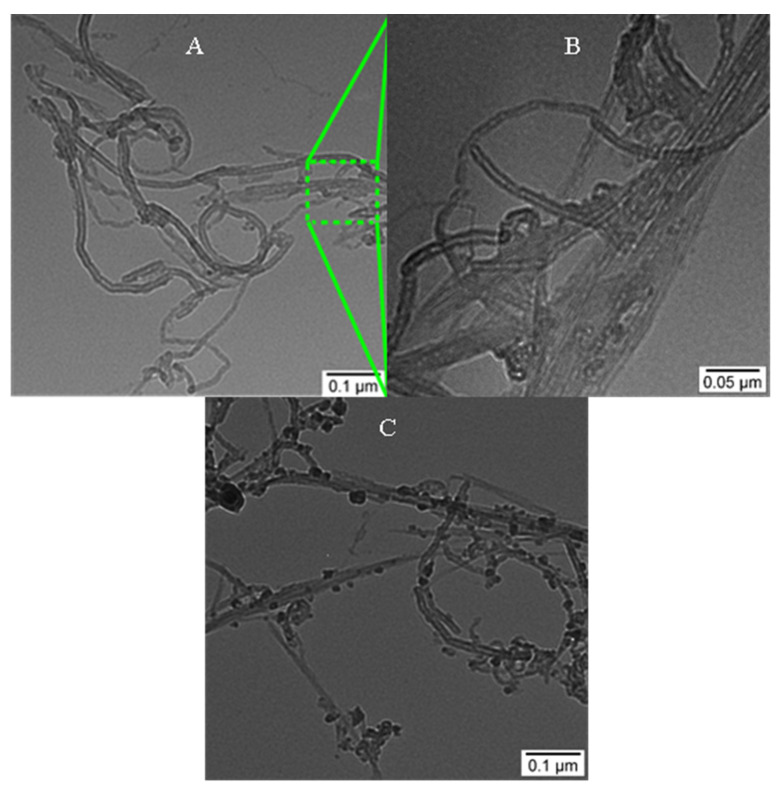
Transmission electron microscopic images of SWCNT-NH_2_/SDS (**A,B**) and SWCNT-NH_2_/DoTAB (**C**). Image B shows the amplification of the area marked in green in the image A. The samples were prepared in a buffer solution NaH_2_PO_4_ 0.01 M/NaCl 0.02 M, pH 3.

This assumption has been confirmed by transmission electron microscopy (see Figure 6 and Figure 7). Figure 6 shows the dispersion of MWCNTs (A and B) and SWCNTs (C and D) in the presence of SDS and DoTAB. These images show that both anionic and cationic surfactants produce a dispersion of non-functionalized CNTs with similar morphology. However, in the case of cationic carbon nanotubes (SWCNT-NH_2_), the anionic surfactant (Figure 7A) clearly causes a poorer dispersion than the cationic ones (Figure 7C). Figure 7B shows the bundled SWCNT-NH_2_ observed in the presence of the anionic surfactant (SDS).

This behavior is also demonstrated from Zeta-potential (ζ) and dynamic light scattering measurements. As is observed in Table 1, non-functionalized CNTs (SWCNT and MWCNT) show similar ζ and size values in the presence of both cationic and anionic surfactants (note that Zeta-potential value is positive for DoTAB and negative for SDS, due to the charge of the surfactants, as was expected). Bearing this in mind, the surface charge on these carbon nanotubes will be similar, as well as the electrostatic repulsions between CNT/surfactant complexes. This means that the dispersion process will be comparable in both systems, as was mentioned above when TEM images were discussed.

In the case of amine-functionalized single walled carbon nanotubes, a change in ζ values is observed depending on the ionic surfactant added, increasing in the presence of DoTAB and decreasing with SDS, compared to the Zeta-potential value of SWCNT-NH_2_ in the absence of any surfactant. Therefore, the dispersion process of the nanotubes will be better in the presence of the cationic surfactants as was in TEM images (see Figure 7). The sizes of SWCNT-NH_2,_ SWCNT-NH_2_/DoTAB and SWCNT-NH_2_/SDS are very similar, only slightly higher in the latter. This agrees with a poor dispersion of the nanosystem.

In relation to the polydispersity index (PDI) values, it is worth mentioning the lower value for the SWCNT-NH_2_/DTAB system and the higher one for SWCNT-NH_2_/SDS compared to the CNTs without any surfactants. This confirms the better dispersion for the amine-functionalized nanotubes in the presence of the cationic surfactant DoTAB.

On the other hand, taking into account that the contribution of the electrostatic interactions is the driving force in the dispersion process, the surfactant concentration needed to obtain a good dispersion depends on the CNT functionalization degree. TEM images of SWCNT/DoTAB and SWCNT--NH_2_/DoTAB (Figure 6C and Figure 7C, respectively) corroborate this fact: functionalized carbon nanotubes ([CNT] = 0.05 g L^−1^) are better dispersed with an aqueous solution of DoTAB 5 × 10^−5^ mol dm^−3^ than the non-functionalized CNTs at the same concentration. This is also confirmed from the ζ, size and PDI values collected in Table 1.

## 3. Material and Methods

### 3.1. Materials

The surfactants studied were hexadecyltrimethyl ammonium bromide (CTAB), dodecyltrimethyl ammonium bromide (DoTAB), octyltrimethyl ammonium bromide (OTAB), and sodium dodecyl sulfate (SDS); all of them purchased from Sigma Aldrich (R grade). The surfactant concentrations used were always lower than the *cmc* to avoid the presence of micelles in the samples. Moreover, the potentiometric technique based on ion-selective membrane electrodes is usually employed to determine monomer surfactant concentrations (see below). Micelles in the solution exert an interaction with nanotubes different than that due to monomers. Therefore, the presence of micelles in the solution must be avoided. The SWCNTs and SWCNT-NH_2_ (both of them 1–5 μm length and 1.5 nm diameter) were obtained from NanoLab Inc. The MWCNTs (1.5 μm length and 10 nm diameter) were supplied by Dropsens S.L. All the solutions were prepared in a buffered media (Na_2_HPO_4_ 0.01 M/NaCl 0.02 M, pH 9). In the case of SWCNT-NH_2_, a buffer solution NaH_2_PO_4_ 0.01 M/NaCl 0.02 M, pH 3 was also used. The choice of these pHs was made in order to have a positive (pH = 3) or neutral (pH = 9) charge on the surface of the SWCNT-NH_2_.

### 3.2. Dispersion of Carbon Nanotubes

The SWCNT, MWCNT, and SWCNT-NH_2_ were dispersed at the desired concentration in an aqueous surfactant solution by sonication during 5 min using a bath sonicator (JP Selecta Ultrasons system, JP Selecta, Barcelona, Spain, 200 W and 50 kHz). The sonication was repeated three times.

### 3.3. Potentiometric Measurements

**Scheme 1:**
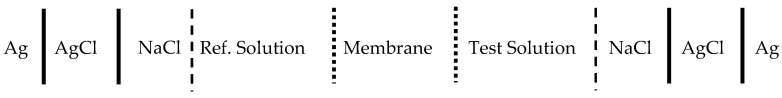
Electrochemical cell based on ion-selective membrane electrodes for potentiometric measurements.

Electromotive forces of solutions containing surfactants and CNTs were measured by using selective electrodes of the different ionic surfactants. Ion-selective membrane electrodes of the cationic (CTAB, DoTAB, and OTAB) and anionic (SDS) surfactants used in this work were synthesized following the procedure previously described [36]. The resulting ion-selective electrodes were conditioned for at least 2 h in a buffered reference solution containing the corresponding surfactant. The surfactant concentrations of these solutions were 2 × 10^−2^ mol dm^−3^ for OTAB, 2 × 10^−3^ mol dm^−3^ for DoTAB, 1 × 10^−4^ mol dm^−3^ for CTAB and 1 × 10^−3^ mol dm^−3^ for SDS. Scheme 1 shows a representation of the cell used in the potentiometric measurements.

Electromotive forces were measured in a custom-built electrometric amplifier using an INA 116 ultra-low input bias current instrumentation amplifier, followed by a unity-gain Sallen–Key low-pass filter [*n* = 4, fc = 15 Hz (−3 db)]. The response was monitored using a Keithley 2110 5½ Digital Multimeter (Keithley Instruments, Cleveland, OH, USA), which was interfaced to a PC through a USB interface with an accuracy of ± 0.1 mV. The temperature was maintained constant by using a glass cell with a thermostated water bath. The concentration of the free surfactant, [S]_f_, was determined using a calibration curve for each ion-selective membrane electrode (Nernst equation).

### 3.4. Transmission Electron Microscopy (TEM)

Transmission electron microscopy samples were prepared as follows: a drop of the aqueous solution containing surfactant and CNTs was deposited on a cooper grid coated with a carbon film. The grid was dried to air at room temperature. The images were visualized with a Philips CM200 electron microscope (Philips, Amsterdam, Netherlands). The concentrations used were [CNT] = 0.05 g L^−1^ (for all the carbon nanotubes used), [DoTAB] = 5 × 10^−5^ mol dm^−3^ and [SDS] = 5 × 10^−5^ mol dm^−3^. All the samples were prepared in buffer phosphate at pH 9 (SWCNT and MWCNT) and 3 (SWCNT-NH_2_).

### 3.5. Zeta-Potential Measurements

Zeta-potential (ζ) experiments were carried out with a Zetasizer Nano ZS Malvern Instrument Ltd. (Malvern, Worcestershire, UK). The values of ζ were determined by measuring the electrophoretic mobility of the samples from the velocity of the particles using a Laser Doppler velocimeter (LDV, (Malvern, Worcestershire, UK). DTS1060 polycarbonate capillary cells were used at 298.1 ± 0.1 K. Each value is the average of at least 5 measurements. The samples used were the same as those utilized in the TEM measurements. In the case of SWCNT-NH_2_, measurements were performed both in the presence and absence of surfactants.

### 3.6. Dynamic Light-Scattering Measurements

The dynamic light-scattering technique was used in order to evaluate the size and the polydispersity index (PDI) of CNTs. The measurements were carried out using a Zetasizer Nano ZS Malvern Instrument Ltd. (Malver, Worcestershire, UK) at 298.1 ± 0.1 K. Samples were laser illuminated with a fixed detection arrangement of 90° to the center of the cell area, and the intensity fluctuation in the scattered light was analyzed. The hydrodynamic diameter (d_H_) was calculated by using the Stokes–Einstein equation. Each result is the average of at least ten measurements. The samples were the same as those used in the Zeta-potential experiments.

## 4. Conclusions

The interaction (adsorption) of commercial ionic surfactants at carbon nanotubes’ surfaces has been investigated using a potentiometric technique with ion-selective membrane electrodes. It has been demonstrated that the adsorption process shows a cooperative character, the hydrophobic interaction’s contribution being the driving force. In fact, the relative free surfactant concentration, for a given [CNT], shows a linear correlation with the logarithm of the critical micellar concentration (*cmc*) that mainly depends on the hydrophobic Gibbs energy contribution. The electrostatic interactions do not play an important role in the adsorption process, but they mainly determined the dispersion of the CNTs by ionic surfactants. The best dispersing agent is the surfactant that produces the highest increment in the surface charge of the nanotubes, thereby increasing the electrostatic repulsions among the CNT/surfactant complexes. However, there is also a contribution of the hydrophobic forces in the dispersion process. In fact, the first step to achieve the CNT unbundling is the formation these CNT/surfactant complexes; that is, the adsorption process. Furthermore, the quantity of the dispersing agent required to optimize the dispersion depends on the degree of CNTs functionalization.

## Figures and Tables

**Table 1:** Zeta-potential (ζ), size (d_H_) and polydispersity index (PDI) values for different carbon nanotubes in the presence and absence of ionic surfactants.

CNT/Surfactant	ζ/mv *	d_H_/nm **	PDI **
SWCNT/DoTAB	4.1 ± 0.2	1180 ± 30	0.455
SWCNT/SDS	−3.9 ± 0.3	1031 ± 25	0.556
MWCNT/DoTAB	1.7 ± 0.1	1483 ± 27	0.598
MWCNT/SDS	−4.5 ± 0.3	1194 ± 32	0.664
SWCNT-NH_2_	13.3 ± 0.4	928 ± 15	0.470
SWCNT-NH_2_/DoTAB	22.5 ± 0.2	920 ± 12	0.391
SWCNT-NH_2_/SDS	8.6 ± 0.3	958 ± 16	0.694

* The experimental **ζ** values are expressed as the mean ± standard deviation (SD) (*n* = 5). ** The experimental d**_H_** and PDI values are expressed as the mean ± SD (*n* = 10).

## Data Availability

Not applicable.

## Supplementary Materials

The following are available online at https://www.mdpi.com/1422-0067/22/2/826/s1, Figure S1: Linear plots of electromotive force versus the logarithm of the free surfactant concentration (calibration curve based on Nernst equation) for all ionic surfactants investigated, Figure S2: Plots of [S]f/[S]0 at different [CNT] versus log (cmc/mol dm-3) for all ionic surfactants and CNTs investigated.
