# Supplementary material for: Potentiometric Study of Carbon Nanotube/Surfactant Interactions by Ion-Selective Electrodes. Driving Forces in the Adsorption and Dispersion Processes
Source: Int J Mol Sci. 2021 Jan 15;22(2):826. doi: 10.3390/ijms22020826 (PMC7830566; doi:10.3390/ijms22020826)
Supplement: Supplementary file 1 [file ijms-22-00826-s001.pdf]

SUPPLEMENTARY INFORMATION

POTENTIOMETRIC STUDY OF CARBON NANOTUBE/SURFACTANT INTERACTIONS BY ION-SELECTIVE ELECTRODES. DRIVING FORCES IN THE ADSORPTION AND DISPERSION PROCESSES

Francisco José Ostos[1], José Antonio Lebrón[1], María Luisa Moyá[1], Eva Bernal[1], Ana Flores[2], Cristian Lépori[3], Ángeles Maestre[2], Francisco Sánchez[1], Pilar López-Cornejo[1]*and Manuel López-López[2]*

[1] Department of Physical Chemistry, University of Seville, c/ Prof. García González 1, 41012 Seville, Spain.

[2] Department of Chemical Engineering, Physical Chemistry and Materials Science. Campus 'El Carmen', Faculty of Experimental Sciences, University of Huelva. 21071 Huelva, Spain.

[3] Institute of Physics Enrique Gaviola (IFEG), National Council of Scientific and Technical Research (CONICET), National University of Córdoba´ (UNC), X5016LAE Córdoba, Argentina.

*Authors to whom all correspondence should be directed. P. López-Cornejo (pcornejo@us.es) and M. López-López (manuel.lopez@diq.uhu.es)

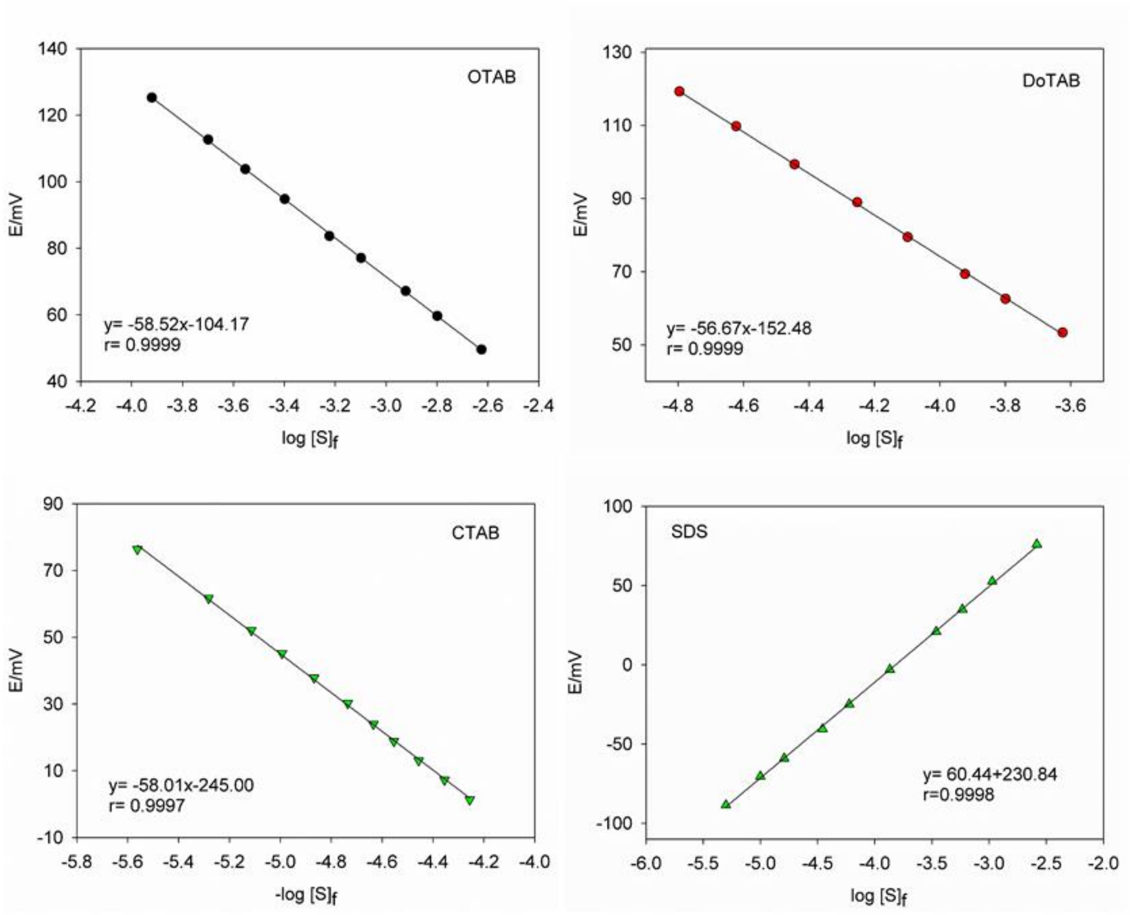

Figure S1. Linear plots of electromotive force *versus* the logarithm of the free surfactant concentration (calibration curve based on Nernst equation) for all ionic surfactants investigated.

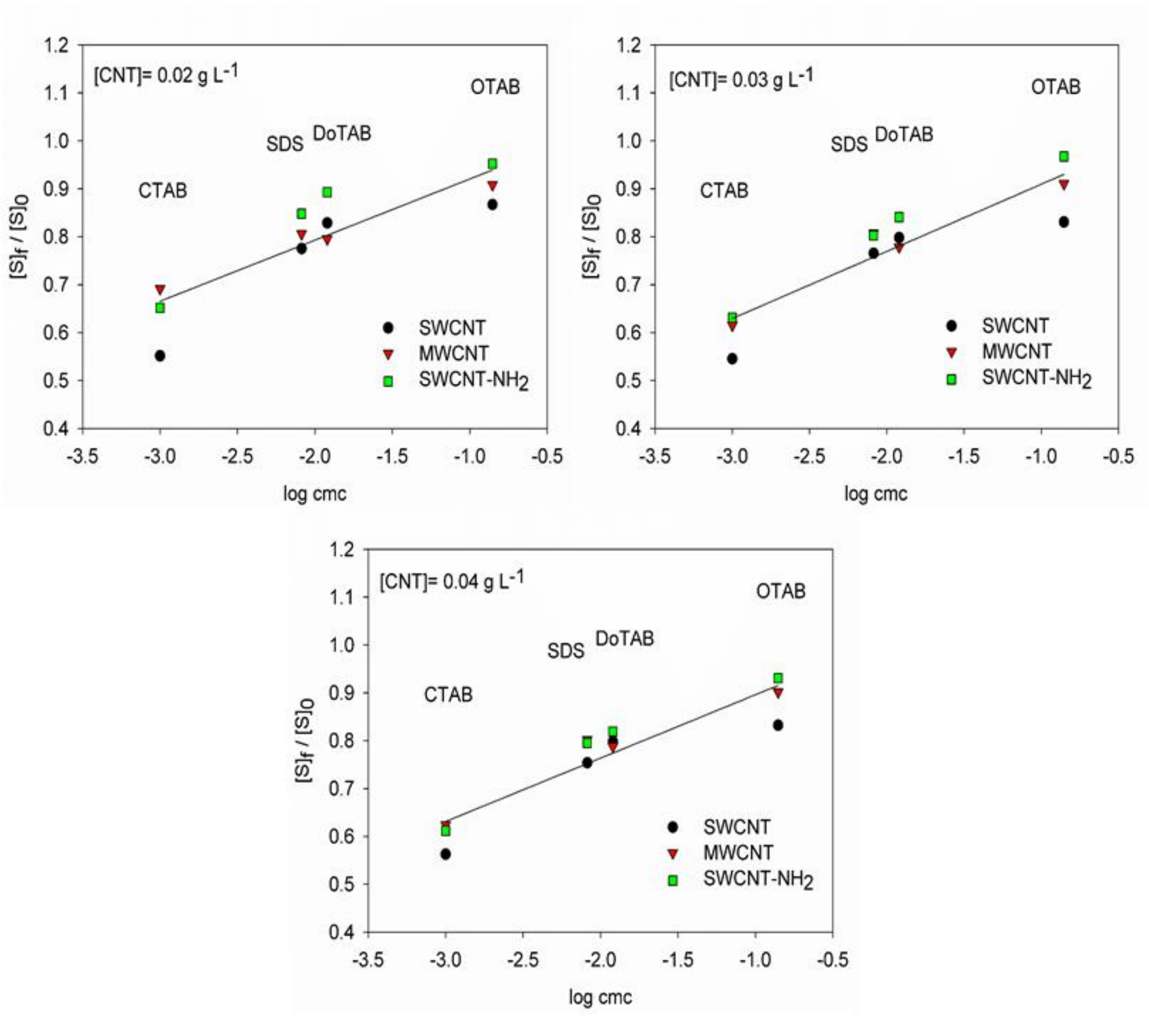

Figure S2. Plots of $[S]_f/[S]_0$ at different [CNT] *versus* log (*cmc*/mol dm^{-3}) for all ionic surfactants and CNTs investigated.
